# Colitis; Preceded by Acute Diarrhœa

**Published:** 1882-01

**Authors:** Mark L. Frey


### VII.—COLITIS; PRECEDED BY ACUTE DIARRHŒA.

REPORTED BY MARK L. FREY, D.V.S.

November 10th, I was called to see a horse, with the following history:

November 9th, at about five A. M., it was first noticed that the animal frequently discharged, after considerable straining, half-fluid, clay-colored fæces of an offensive odor; also, refused to eat, eye dull, great thirst and rapid emaciation and debility.

The following medicine was administered by the groom:

℞

Oleum Ricini, ℥ iv.

Tincture Opii, ℥ ii.

Oleum Cini, Oi.

This was given twice that day, followed by warm water injections per rectum. The following morning, the owner seeing that the animal was apparently worse, sent for me.

Upon examination I ascertained that the discharged fæces were tinged with blood and pus; there was considerable straining at the anus and of the rectum upon attempts to evacuate the bowels. There was pain in the abdominal region; dullness of the eye, great thirst, urine frequently discharged, of a high color and of high specific gravity; malassimilation of food, and a general debility. The urine contained large quantities of urea, due to the rapidity of tissue metamorphosis.

Diagnosticated Colitis, preceded by an acute diarrhœa.

Treatment:

℞
Pulvis Opii, et.
Pulvis Galli, et.
Cupri Sulphatis, āā, ℥ ii.
Mx. et. fiat in chart No. 2.

Sig. twice daily by mouth.

Also:

℞

Pulvis Opii.

Plumbi Acetatis, āā ℥ ij.

Aquæ Amylum, Oij.

Mx. sig. ½ to be thrown up per rectum, by the syringe, the second half to be used at the end of one hour.

The following day ordered for the debility:

℞

Pulvis Opii, ℨ ss.

Spiritus Frumenti, ℥ ij.

Mx. sig.; twice daily.

The above plan of treatment was faithfully carried out, and the animal made a speedy recovery.

